# Can Genetic Algorithms Be Used for Real-Time Obstacle Avoidance for LiDAR-Equipped Mobile Robots?

**DOI:** 10.3390/s23063039

**Published:** 2023-03-11

**Authors:** Zoltán Gyenes, Ladislau Bölöni, Emese Gincsainé Szádeczky-Kardoss

**Affiliations:** 1 Department of Computer Science, University of Central Florida, 4328 Scorpius St., Orlando, FL 32816, USA; 2 Department of Control Engineering and Information Technology, Faculty of Electrical Engineering and Informatics, Budapest University of Technology and Economics, Műegyetem rkp. 3., H-1111 Budapest, Hungary

**Keywords:** genetic algorithm, obstacle avoidance, velocity obstacles

## Abstract

Despite significant progress in robot hardware, the number of mobile robots deployed in public spaces remains low. One of the challenges hindering a wider deployment is that even if a robot can build a map of the environment, for instance through the use of LiDAR sensors, it also needs to calculate, in real time, a smooth trajectory that avoids both static and mobile obstacles. Considering this scenario, in this paper we investigate whether genetic algorithms can play a role in real-time obstacle avoidance. Historically, the typical use of genetic algorithms was in offline optimization. To investigate whether an online, real-time deployment is possible, we create a family of algorithms called GAVO that combines genetic algorithms with the velocity obstacle model. Through a series of experiments, we show that a carefully chosen chromosome representation and parametrization can achieve real-time performance on the obstacle avoidance problem.

## 1. Introduction

Most current-generation mobile robots are used in warehouses and factories [1,2,3,4] and operate in areas physically separated from human workers. Future robots deployed in public spaces will need to move reliably without colliding with humans or other obstacles. For this, they need to solve the problem of *navigation in dense dynamic environments*, which can be broadly formulated as the task of finding the set of commands that takes a mobile robot from a start position to a desired destination in minimum time without colliding with static or mobile obstacles. The difficulty of this problem stems from its real-time nature [5], the uncertainty in sensing the environment [6,7], and the fact that human agents do not follow predictable trajectories [8,9].

A tension exists between the complexity of the optimization problem and the requirement of replanning the robot trajectory on a very short deadline [10,11]. In many papers in the literature, this tension was resolved by foregoing advanced optimization algorithms in favor of techniques that are easier to deploy under tight time constraints. However, with the advancements in computing architectures and optimization, new classes of algorithms might become suitable for real-time deployment. In this paper, we consider genetic algorithms (GAs), optimization techniques, which historically were considered too compute-intensive for real-time decisions, although usable for path-planning in offline scenarios.

Our objective is to investigate whether, with our current level of hardware and algorithmic knowledge, GAs can be deployed for real-time path planning and obstacle avoidance and what are the benefits and limitations of such a deployment. In particular, we combine the improved velocity obstacle (VO) method with a GA, and explore different data representations, parametrizations, and recombination algorithms in the search for a competitive real-time solution. The main contributions of this paper can be summarized as follows:

- We propose a mobile robot path-planning algorithm with dynamic obstacle avoidance based on a combination of genetic algorithms and velocity obstacles.
- We demonstrate that by a judicious choice of data representation, parameters, and recombination algorithms, GAs can achieve a real-time decision speed in this setting.
- Through a set of extensive experiments we compare variants of the proposed approach with baselines, considering the running time and the executed path of the mobile robot.

The rest of the paper is structured in the following way: Section 1 presents previous research results considering global and local motion planning algorithms for mobile robots. Section 3 describes the foundations of the velocity obstacles motion planning method that forms the base of our proposed approach. Section 4 explains the steps of the introduced genetic algorithm-based velocity obstacles motion planning method while Section 5 describes the experimental study that investigates the performance and computational cost of the proposed approach. We conclude in Section 6.

### Related Work

Based on the level of knowledge about the environment, motion planning for robots can be classified into global and local algorithms.

Offline *global motion planning* methods assume that complete information about the environment exists a priori. Such information is usually only available for static environments. Examples of global motion planning methods include a hybrid A* [12], rapidly-exploring random tree (RRT) [13,14,15].

In contrast, *local motion planning* techniques consider only information about the immediate neighborhood of the robot, information that is usually accessible from the robot’s own sensors. A typical example is the artificial potential field method, where obstacles create potential fields acting as repulsive virtual forces while the destination generates an attractive virtual force. The vector sum of these forces allows the planner to calculate the velocity vector of the robot at every sampling interval [16,17,18,19,20]. While initially proposed for static environments, the potential field model was also extended for dynamic obstacles, such as robot soccer [20].

The dynamic window approach (DWA) is a widely used motion planning method for mobile agents. This motion planning method can be used to generate real-time solutions. Non-holonomic constraints, such as limited turning ability can be considered and the method can be used in dynamic environments to generate a collision-free path [21,22]. The DWA algorithm was used recently for the solution of the motion planning problem of the forklift-automated guided vehicle [23].

Another well-known local motion planning algorithm is the velocity obstacle (VO) algorithm [24]. The VO calculated by the algorithm is the locus of the velocity vectors that would cause a collision between the agent and the obstacles in a future step. At every sampling step, the robot selects a velocity vector that would result in a collision-free motion, following a specific strategy. In the ’to goal’ (TG) strategy, the agent selects the largest velocity vector in the line between the agent and the goal. The maximum velocity (MV) strategy selects the maximum velocity within a certain angle to the path to the goal. Various other strategies are possible, including strategies that temporarily move away from the goal in order to avoid collisions. The VO technique had been extended to a range of scenarios including differential-driven robots [25,26], ships [27], UAVs (unmanned aerial vehicles) [28], and multi-robot collision avoidance [29,30,31]. Although the VO method is basically developed for mobile robots, it can also be applied to robotic manipulators [32]. An energy-efficient algorithm was also introduced for UAVs with limited battery capacity in multi-robot scenarios [33,34].

One of the first methods that proposed the use of GAs in path planning was [35], where the objective was to reach a goal in an environment with no obstacles. Ref. [36] used GAs in a global path planning setting to find an optimal path in a large-scale grid environment. Reference [37] combined GAs with a probabilistic roadmap model for path planning in a static environment. Multi-objective genetic algorithms, such as NSGA (non-dominated sorting genetic algorithm) can also be used for these tasks [38]. Reference [39] used the GA for multiple agent tasks. GA was combined with recurrent neural networks in [40]. Most of these research projects assumed a static environment. While projects such as [41,42] demonstrated that the GA model can be adapted to dynamic environments with moving obstacles, it was not possible to run the algorithms in real time.

## 2. Problem Formulation

Consider the following problem: Given a disk-shaped robot *A* that moves in a 2D workspace, its position is defined as the center of the circle, and it is denoted by a vector $p_{A}={\left(x_{A},y_{A}\right)}^{T}$. The radius of *A* is $r_{A}$. Its velocity vector is denoted by $v_{A}={\left({\dot{x}}_{A},{\dot{y}}_{A}\right)}^{T}$. The agent is modeled as an omnidirectional robot such that its motion capabilities are restricted by some constraints (e.g., maximal velocity $v_{max}$).

The environment contains *m* obstacles. They are denoted by $B_{i}$, i=1…m. The obstacles are also modeled by circles with radius $r_{\mathrm{Bi}}$. The position of $B_{i}$ is the position of its center $p_{\mathrm{Bi}}={\left(x_{\mathrm{Bi}},y_{\mathrm{Bi}}\right)}^{T}$. The obstacles move with velocity $v_{\mathrm{Bi}}={\left({\dot{x}}_{\mathrm{Bi}},{\dot{y}}_{\mathrm{Bi}}\right)}^{T}$. (Note that a static obstacle is modeled as a moving obstacle such that $v_{B}=0^{T}$).

An often-used technique is to model robot *A* by a single point by enlarging the radius of the obstacles by $r_{A}$. The task is to plan the motion for this point such that it does not touch the enlarged obstacles.

The robot has a target. The position of this target is denoted by $p_{g}={\left(x_{g},y_{g}\right)}^{T}$. During the navigation of the robot, in each sampling time moment, the task is to select such a velocity vector $v_{A}$, which ensures collision-free motion, such that the robot is able to reach its goal $p_{g}$ as fast as possible.

## 3. Background

Our approach builds on the velocity obstacle (VO) algorithm [24], whose objective is to select a velocity vector that generates no collision for the agent if the position and velocity data of the obstacles are known or measurable at the time of decision making.

The velocity obstacle cone $VO_{i}$ is the set of all velocity vectors of agent *A* that would result in a collision with the obstacle $B_{i}$ at a later time:

(1) $$VO_{i}=\left\{\;v_{A}\;|\;\exists \;t:A\left(p_{A}+v_{A}t\right)\;\cap \;B_{i}\left(p_{\mathrm{Bi}}+v_{\mathrm{Bi}}t\right)\neq 0\right\}$$

In this equation, A(p) and $B_{i}\left(p\right)$ are the set of points in the workspace which are occupied by the robot (respectively obstacle *i*) if its position is p. (1) says that applying a robot velocity vector $v_{A}\in VO_{i}$ will result in a collision between *A* and $B_{i}$ at time *t*. (As an assumption, the velocities of the obstacles and the robot are unchanged until *t*). On the other hand, the $v_{A}\notin VO_{i}$ velocity selection ensures that *A* and $B_{i}$ will not collide until they do not change their velocities $v_{A}$ and respectively $v_{Bi}$.

Considering all the obstacles, the complete velocity obstacle *VO* is the union of the $VO_{i}$ sets:

(2) $$VO=\cup_{i=1}^{m}\;VO_{i}$$

The reachable velocities set RV is the set of all feasible velocity vectors $v_{A}$ that can be reached by the robot by the sampling time considering its motion capabilities (e.g., bounded inputs). The key step in the VO method is the calculation of the reachable avoidance velocities (RAV) set, which can be calculated by subtracting from RV the VO set, i.e., determining the robot velocities that are feasible and will not cause any collision. These are the velocities from which the robot can choose to obtain a collision-free path. A frequent approach is to discretize the RAV set by overlaying a grid on the RAV area.

**Figure 1:**
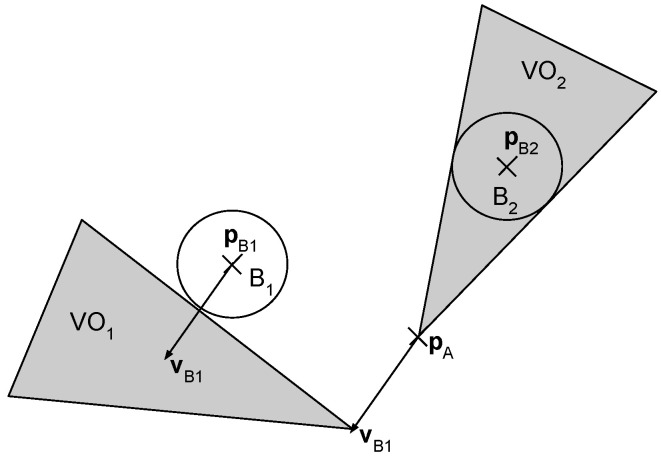
An example of the VO method for a robot at position $p_{A}$ navigating an environment consisting of a moving obstacle $B_{1}$ at position $p_{B1}$ with velocity $v_{B1}$ and a static obstacle $B_{2}$ at $p_{B2}$. The gray area illustrates $VO=VO_{1}\cup VO_{2}$.

Figure 1 shows the application of the VO method for the case of a robot navigating an environment with a static and a mobile obstacle. In the case of the static disk-shaped obstacle $B_{2}$, the $VO_{2}$ is a cone in the plane, such that its vertex is at $p_{A}$ and its sides are tangent to the obstacle’s circle. For the moving obstacle $B_{1}$, the cone has to be translated by the velocity vector $v_{B1}$ of the obstacle to obtain the corresponding $VO_{2}$ set.

## 4. Genetic Algorithm-Based Velocity Obstacle Method

The primary challenge of techniques derived from the VO model is the selection of the velocity vector. All the vectors from the RAV set can be part of a collision-free trajectory, but they are not equally useful for the robot in its goal to reach the destination. The original VO paper [24] proposed two simple single-shot heuristics for selecting the velocity vector in real-time settings: the To-Goal and the Maximum Velocity strategies. These strategies might not choose the optimal velocity vector for reaching the destination, especially in crowded environments, but they are computationally inexpensive and provide satisfactory solutions in many scenarios.

The idea for our genetic algorithm-based velocity-obstacle (GAVO) model is to calculate the VO and RAV areas and use a GA to evolve a solution that is feasible (i.e., it is part of the RAV) while aiming to optimize the performance criteria desirable for the user.

The search space of the possible solutions is infinitely large. While this can be discretized by applying a grid to the RAV area, performing a high-resolution grid search can make the process prohibitively expensive. The GA algorithm allows us to search areas outside the grid and direct the search toward promising areas. However, GAs can also be computationally expensive. To achieve a good performance in the limited time allotted to the robot, the GAVO algorithm needs to make intelligent decisions about pre-filtering solutions, the choice of the encoding as well as the recombination and mutation operators.

### 4.1. Filtering Obstacles

One way to improve the performance of the GAVO algorithm is by simplifying the calculations involving the sampling. Let us consider that the robot is planning ahead for a time window $T_{max}$. There are two types of obstacles that the robot can ignore in its path planning: (a) the obstacles which cannot be reached in time $T_{max}$ and (b) the obstacles which are going to be sufficiently far away even when the robot is at the closest distance to them.

To filter out these obstacles from consideration we start by calculating the time when the robot and the obstacle will be closest to each other

(3) $$t_{min_{A,\mathrm{Bi}}}=\frac{-\left(p_{A}-p_{\mathrm{Bi}}\right)\left(v_{A}-v_{\mathrm{Bi}}\right)}{\left|\right|v_{A}-v_{\mathrm{Bi}}\left|\right|},$$

and the minimum distance at that moment:

(4) $$d_{min_{A,Bi}}=\left|\right|\left(p_{A}+v_{A}t_{min_{A,Bi}}\right)-\left(p_{\mathrm{Bi}}+v_{\mathrm{Bi}}t_{min_{A,Bi}}\right)\left|\right|,$$

Having these values we can ignore those obstacles $B_{i}$ for which $t_{min_{A,Bi}}$ is larger than $T_{max}$ or $d_{min_{A,Bi}}$ is larger than a predefined safe distance $d_{max}$.

### 4.2. Fitness Value

In GAVO, the individuals subject to selection are a representation of a velocity vector, while their fitness must reflect the performance of the given vector in fulfilling the objectives of the robot of staying safe and moving toward the destination. Accordingly, the fitness function we designed reflects the two components of speed and safety.

The speed component measures the progress toward the target position:

(5) $$GO\left(v_{i}\right)=\frac{r_{i}\cos\Delta \theta_{i}}{v_{max}}$$

where $v_{max}$ is the maximum velocity that the agent can reach considering the kinematic constraints. $r_{i}=||p\left(v_{i}\right)-p_{A}\left|\right|$ is the length of the velocity vector ($\left|\right|v_{i}\left|\right|$) while $\Delta \theta_{i}=\theta_{rg}-\theta_{rv_{i}}$ is the difference between the angle of the goal ($\theta_{rg}$) and the angle of the velocity vector ($\theta_{rv_{i}}$).

The safety component of the fitness measures whether there is a risk of collision. It is sufficient to consider only the closest VO. Furthermore, a situation where the robot cannot reach even the closest obstacle during the considered time interval is considered perfectly safe, and keeping farther away would not improve safety. These considerations lead us to the following expression:

(6) $$SA\left(v_{i}\right)=\min\left(1,\frac{\min_{v_{VO}\in VO}\left|\right|v_{i}-v_{VO}\left|\right|}{v_{max}T_{max}}\right)$$

Finally, we combine the speed and safety components of the fitness function as a weighted average, with the parameter β capturing the importance of the progress toward the destination. For velocity vectors that are not in the RAV set, we artificially set the fitness to zero:

(7) $$f\left(v_{i}\right)=\left\{\begin{array}{ll} \left(1-\beta \right)\;SA\left(v_{i}\right)+\beta \;GO\left(v_{i}\right) & \mathrm{if}\;v_{i}\in RAV\\ 0 & \mathrm{otherwise} \end{array}\right.$$

### 4.3. Recombination Method

In implementing the genetic algorithm component of GAVO, we experimented with three different recombination methods, all of them designed to take advantage of the problem domain and the chosen representation model. For all three techniques, the selection of the chromosomes chosen for recombination is performed using stochastic universal sampling [43].

In the *linear recombination* method, the new velocity vector is a random linear combination of the odd and even parents $v_{1}$ and $v_{2}$

(8) $$v_{\mathrm{new}}=v_{1}+\kappa \;\left(v_{2}-v_{1}\right)$$

where κ is a random parameter with a pre-defined range and it is the same for all of the alleles in the recombination.

In the *intermediate recombination* method, we use two different random parameters $\kappa_{x}$ and $\kappa_{y}$ for the two components of the new vectors:

(9) $$v_{newX}=v_{1x}+\kappa_{x}\;\left(v_{2x}-v_{1x}\right)$$

(10) $$v_{newY}=v_{1y}+\kappa_{y}\;\left(v_{2y}-v_{1y}\right)$$

Finally, in the *polar coordinate recombination* method, the recombined vector is a result of a separate combination of the angle and radius in a polar coordinate representation of the two parent vectors:

(11) $$v_{newX}=(\min\left(r_{1},r_{2}\right)+\kappa_{1}\;|r_{1}-r_{2}|)\cdot \cos(\min\left(\theta_{1},\theta_{2}\right)+\kappa_{2}\;|\theta_{1}-\theta_{2}\left|\right)$$

(12) $$v_{newY}=(\min\left(r_{1},r_{2}\right)+\kappa_{1}\;|r_{1}-r_{2}|)\cdot \sin(\min\left(\theta_{1},\theta_{2}\right)+\kappa_{2}\;|\theta_{1}-\theta_{2}\left|\right),$$

where, $|r_{1}-r_{2}|$ and $|\theta_{1}-\theta_{2}|$ are the absolute values of the differences.

**Figure 2:**
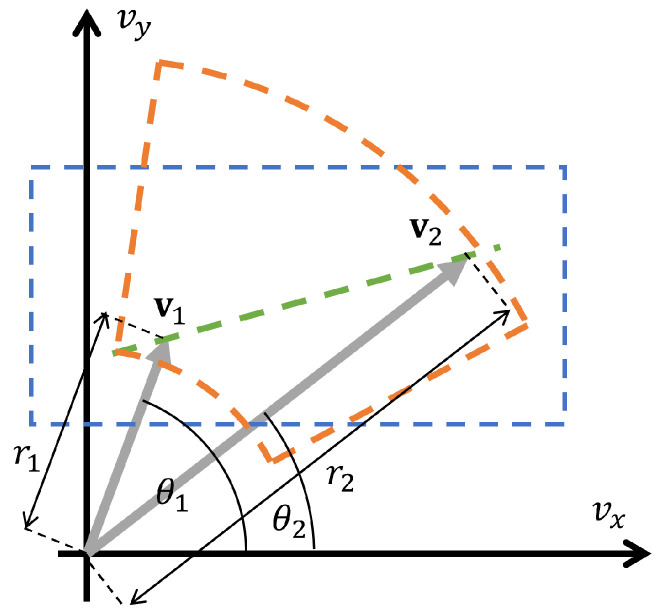
Illustrating the three recombination techniques for the velocity vectors $v_{1}$ and $v_{2}$. The green line shows the possible outcomes of the linear recombination, the blue area shows the possible endpoints of the intermediate recombination, while the orange area shows the possible endpoints of the polar coordinate recombination method.

Figure 2 illustrates the three recombination methods.

### 4.4. Mutation

The mutation technique we use is based on adding random values $\gamma_{1}$ and $\gamma_{2}$ drawn from a predefined range to the two components of the velocity vector representing the chromosome.

(13) $$v_{newX}=v_{1x}+\gamma_{1}$$

(14) $$v_{newY}=v_{1x}+\gamma_{2}$$

In scenarios where recombination is being used, the mutation is applied to a small random fraction of the resulting vectors. In the scenario where no recombination is used, the mutation is applied to every phenotype.

## 5. Experiments and Results

In this section, we describe experiments that compare variations of the GAVO approach along the dimensions of the quality of the solution found and computational cost. We are particularly interested in whether the approach can reach the optimal solution (or come very close to it) and whether the technique is suitable for real-time operation. For our purposes, we define as real-time a path planner which can reach decisions faster than the sensors’ acquisition rate, which, in the case of the robot architecture we are considering is 10 Hz corresponding to a 100 ms time left for the computation.

The variations of the GAVO algorithm we consider are as follows:

- GAVO-1D: uses the 1D recombination method with κ∼[−0.25,1.25] and mutation rate 1/N.
- GAVO-2D: uses a 2D recombination method with $\kappa_{x}∼\left[-0.25,1.5\right]$, $\kappa_{y}∼\left[-1,1\right]$ and mutation rate 1/N.
- GAVO-POLAR: uses the polar recombination method with $\kappa_{1}∼\left[-0.15,+0.15\right]$, $\kappa_{2}∼\left[-5^{\circ },5^{\circ }\right]$ and mutation rate 1/N.
- GAVO-MUT: mutation only—uses the mutation at a rate of 100% and does not perform recombination.

These variants have been tested with different parameters. The number of individuals in each generation *N* was chosen from the range of [20…200]. The generation gap parameter GAP describing the level of elitism in the strategy was chosen from the range [5…10]. To ensure a consistent comparison between the GAVO variants, in every experiment, every variation was started with the same initial population. For the sake of consistency, in all our experiments, we run the experiment for 100 generations, although only a subset of these generations would usually fit into the available time of 100 ms.

These algorithms were compared with two variations of the original velocity-obstacle algorithm: VO-GridSearch [44] performs grid search over the various locations outside the VO areas in the pursuit of the same fitness function, while VO-RandomSearch performs a random search.

For all algorithms, the experimental methodology involved (a) presenting the algorithm with a specific scenario (b) running the algorithm up to a specific point and (c) evaluating the best movement solution found by the algorithm up to that point. For the single shot algorithms VO-GridSearch and VO-Random, there is a single value; while for the GAVO approaches, we record the best available solution at every generation.

The algorithms were implemented in MATLAB 2021a. To keep the computational time measurements consistent, all the experiments were run on a computer with an Intel i5-3320M CPU with 8GB of memory. The memory was found sufficient for the computation making the experiments CPU-limited. No significant disk activity was observed during the experiment and no GPU acceleration was used. The capabilities of this system are comparable with a typical onboard computer of mobile robots.

### 5.1. A sparse Environment

**Figure 3:**
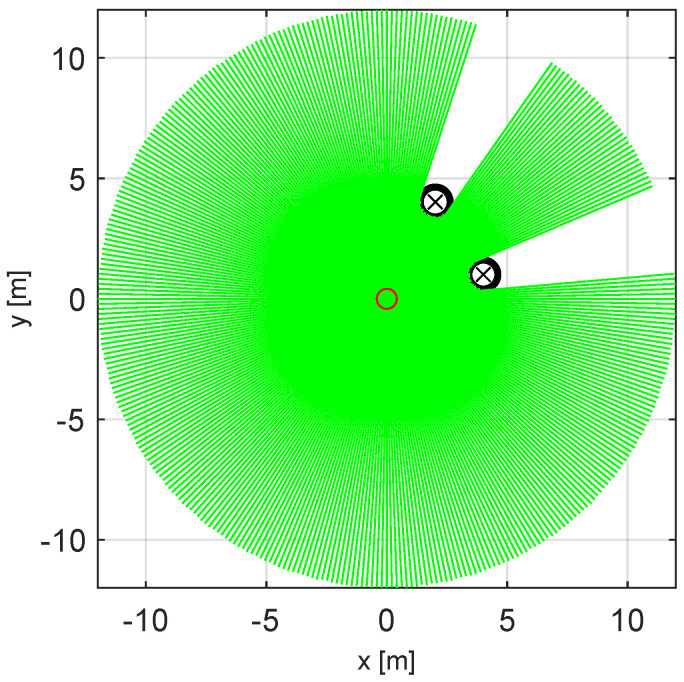
LiDAR sensor data simulation from the workspace of the agent. The agent is in the origin, represented by the red circle, there are two obstacles in the workspace, represented by black circles, and the estimated positions of the obstacles are shown by black x−s. The maximum range of the LiDAR sensor is 12 m, and the resolution is 0.5${}^{\circ }$.

**Figure 4:**
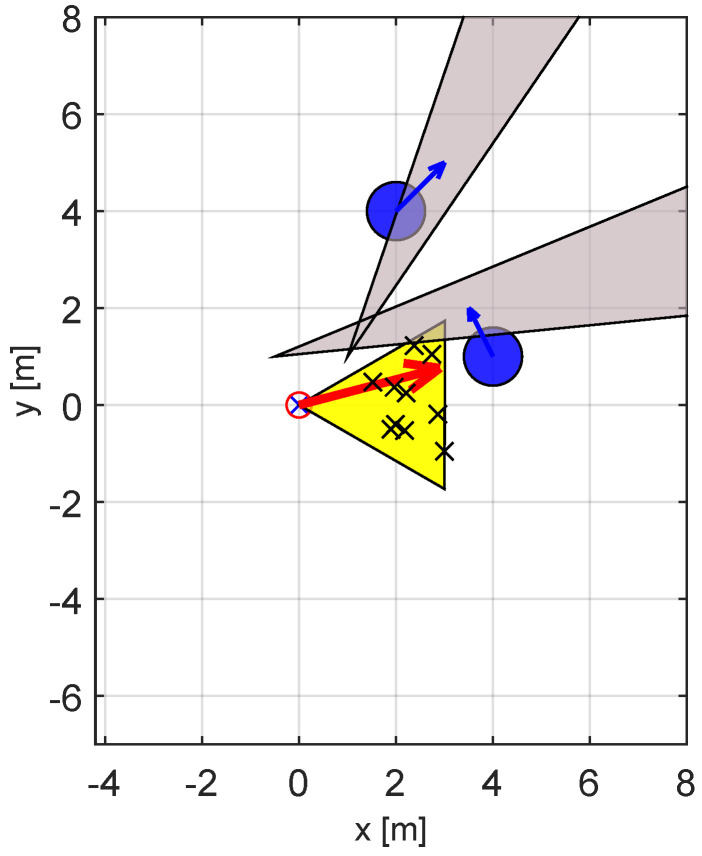
VO−type diagram of the sparse scenario. The robot is positioned in the origin and is denoted by a red circle. The yellow area represents the reachable available velocities, with the red arrow being the optimal velocity corresponding to the fastest solution. The blue disks represent the obstacles with their velocities, while the gray triangles are the corresponding VOs. The x-marks distributed within the area of reachable velocities are the initial population of the GAVO variants. The fact that the area of reachable velocities only minimally overlaps with any VO shows that this is an “easy” scenario.

The first set of experiments was performed in a comparatively easy scenario that takes place in a sparse environment with only two obstacles. We assume that the robot uses LiDAR-based sensing as shown in Figure 3, with a particle filter-based estimator [45]. Figure 4 shows the scenario, the VOs, and the initial population used by the GAVO variants.

**Figure 5:**
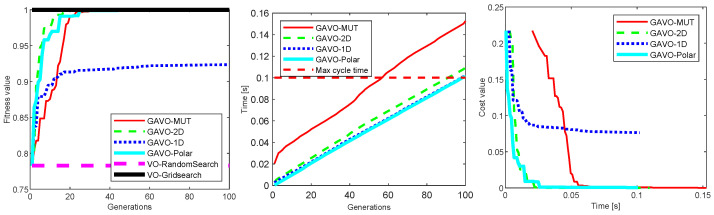
Results of experiments with the sparse scenario for the variations of GAVO with N=20 and GAP=10. VO-RandomSearch and VO-GridSearch serve as baselines that do not change with the generation. (**left**) Evolution of the fitness value with the generations, (**center**) time for a specific number of generations, (**right**) the trade-off between wall clock time and cost defined as 1-fitness.

One of the first considerations for a genetic algorithm is the evolution of fitness with the generations. While the use of elitism in the population guarantees that the maximum fitness will not decrease, there is no guarantee that an optimal solution will be reached in a finite time. Figure 5-left compares the fitness values for different GAVO variants for a scenario using a population size of N=20 and GAP=10. The algorithms started from the same population (the one in Figure 4), and were run for 100 generations. The figure also contains the fitness reached by VO-RandomSearch, which serves as a baseline, and the VO-GridSearch which, due to its exhaustive search nature, will serve as our optimal baseline. We find that there is a significant difference between the different GAVO variations. Except for GAVO-1D which did not find the optimum in the experiment, all the other algorithms find the optimal solution within 33 generations. The best-performing algorithm was GAVO-2D, which reached the best solution in 23 generations.

As the robot needs to make movement decisions in real time, counting the generations in the GAVO optimization provides only part of the answer. We also need to investigate how long a certain number of generations take. This number obviously varies with the speed of the computer and the size of the population. We also speculate that it also varies for the different GAVO variants. Figure 5 describes the time at different generation points for the optimization. The figure also shows the important time point of the maximum cycle time which in our current setup is 0.1 s (corresponding to the 10 Hz sampling rate). In general, it is not practical to run the algorithm longer than this time. As expected, we find that the time spent increases roughly linearly with the number of generations. The fastest versions are GAVO-1D and GAVO-Polar, which are essentially indistinguishable, with GAVO-2D being slightly slower and GAVO-MUT being significantly slower. A way to interpret these results is that to remain in real-time we can run 98 generations of GAVO-1D and GAVO-Polar, about 93 for GAVO-2D and about 56 for GAVO-MUT. Note that this is sufficient for GAVO-Polar, GAVO-MUT, and GAVO-2D to reach the optimal values.

Finally, another perspective is to consider the time spent in optimizing versus the cost defined as 1.0-fitness. In a real-time system, a system might often prefer a satisfying (suboptimal, but “good enough”) solution that can be obtained quickly to an optimal solution that requires significantly more computation. Figure 5 shows the trade-off between the wall clock time and the cost. We notice that the optimal result can be reached by GAVO-2D and GAVO-Polar in roughly the same time (about 26 ms), while GAVO-MUT requested about 55.4 ms to reach the same result. The figure also shows that very close results, of cost of about 0.04 can be reached even faster, in about 10 ms using GAVO-2D or GAVO-Polar.

**Figure 6:**
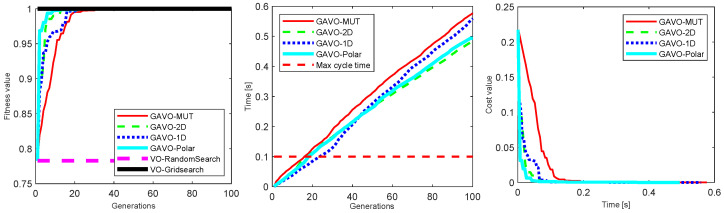
Results of experiments with the sparse scenario for the variations of GAVO with N=100 and GAP=10. VO-RandomSearch and VO-GridSearch serve as baselines that do not change with the generation. (**left**) Evolution of the fitness value with the generations, (**center**) time for a specific number of generations, (**right**) the trade-off between wall clock time and cost defined as 1-fitness.

The second series of experiments repeated the same experiments for a larger population of N=100 with the results shown in Figure 6. We find that the overall shape and trends in the results are the same, however, the concrete values shifted, which has significant implications for the real-time performance of the system. Due to the larger population, all the GAVO variants needed a smaller number of generations to reach the optimal solution. The most significant shift occurred for GAVO-1D which in the smaller population could not reach the optimum in 100 generations, but in this case, it reached it in 22 generations, a better value than GAVO-MUT. The variant that required the lowest number of generations to reach the optimum remained GAVO-2D. The ordering for the time needed for a given number of generations (Figure 6-center) remained the same, however, the times are significantly longer, corresponding to the larger population. This means that we can run a much smaller number of generations before we run into the real-time constraint. For the mutation-only strategy GAVO-MUT, the system cannot reach the optimum before the time runs out. The other three variants can reach the optimum before the maximum cycle time expires.

Finally, the wall-clock time/cost trade-off also changed. While GAVO-POLAR and GAVO-2D remain the best choice, the GAVO-1D strategy is quite close to them, with the GAVO-MUT strategy achieving a significantly worse trade-off.

**Figure 7:**
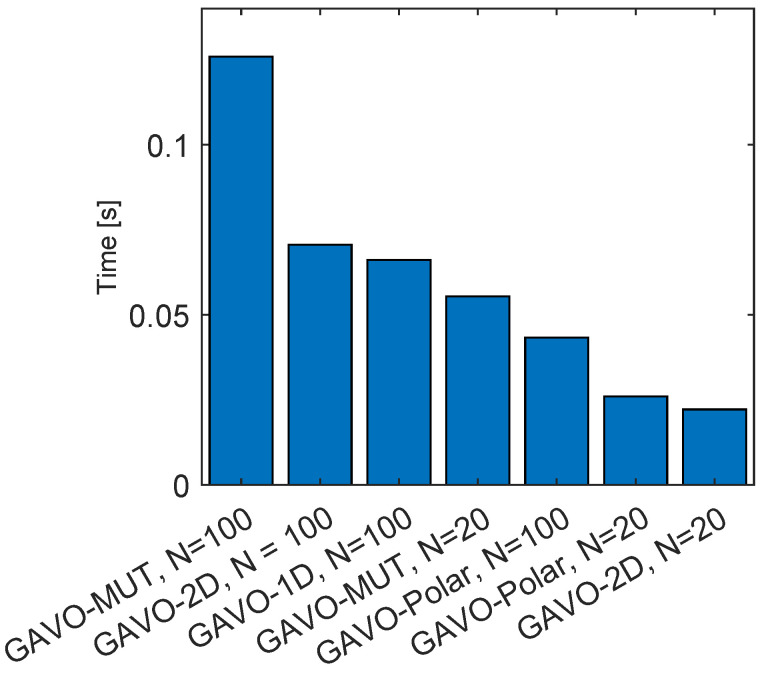
Comparison of the wall-clock time to the optimal solution for GAVO variants with different parametrizations.

The GAVO variants with different population sizes are all possible alternatives for a real-time obstacle avoidance system. Figure 7 compares them for the time it takes for the system to reach the optimal solution. We found that all the combinations considered, except the GAVO-MUT variant with N=100 which takes longer than the maximum cycle time to reach the optimum, and GAVO-1D with N=20, which did not reach the optimum in our experiments and, thus, is not present in this figure. The best time was reached by GAVO-2D with N=20. These values are better compared to previous GA-based algorithms for path planning in dynamic environments, such as [41,42]); however, a direct comparison is not possible as the computational power of typical machines had significantly increased in the decade since the development of those algorithms.

**Figure 8:**
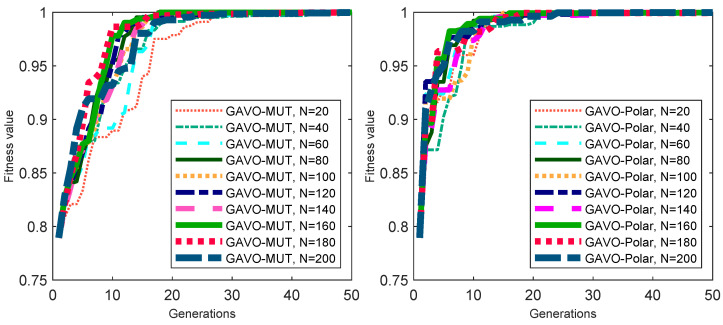
Results of experiments with the sparse scenario for the various number of individuals *N*. (**left**) Evolution of the fitness value with the generations using the GAVO-MUT method, (**right**) Evolution of the fitness value with the generations using the GAVO-Polar method.

We also examined the impact of varying the number of individuals (*N*) on fitness value outcomes in both GAVO-MUT and GAVO-Polar methods. Figure 8-left depicts the fitness value outcomes of the GAVO-MUT method using different values of *N*. Notably, the results demonstrate that the fitness value outcomes are not significantly influenced by the number of individuals in use; rather, the optimal fitness value can be achieved within approximately 20 generations. While the number of individuals does not substantially affect the number of generations required to attain the optimal fitness value, it does play a critical role in the overall running time of the algorithm. As depicted in Figure 7, a real-time solution cannot be attained using 100 individuals. Thus, the GAVO-MUT method necessitates the use of even fewer individuals to attain acceptable running time outcomes.

Figure 8-right presents the outcome of the GAVO-Polar method. In most instances, the optimal solution can be obtained within 20 generations; however, if *N* is set to a smaller value (20), it takes 38 generations to reach the optimal solution. Notably, based on the outcomes depicted in Figure 5 and Figure 6, the GAVO-Polar method can achieve a real-time solution in all scenarios. Therefore, the user can define the number of individuals based on other relevant considerations, and the optimal solution can be reached in all cases.

**Figure 9:**
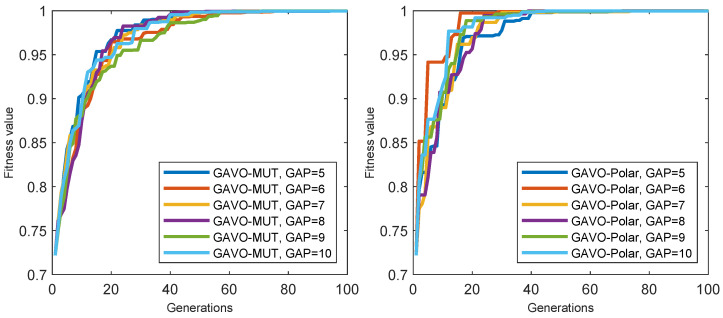
Results of experiments with the sparse scenario for the different GAP parameters. (**left**) Evolution of the fitness value with the generations using the GAVO-MUT method, (**right**) Evolution of the fitness value with the generations using the GAVO-Polar method.

We conducted an investigation into the impacts of varying the GAP parameters of [5; 10] on the fitness values using both the GAVO-MUT and GAVO-Polar methods. The results are presented in Figure 9. The curves of the lines in the GAVO-MUT method (left side) are similar across different GAP values; however, it is evident that the optimal fitness value is achieved after more generations compared to the GAVO-Polar method (right side). Therefore, the GAVO-Polar method appears to be a superior choice for the velocity selection task.

### 5.2. Crowded Environment

**Figure 10:**
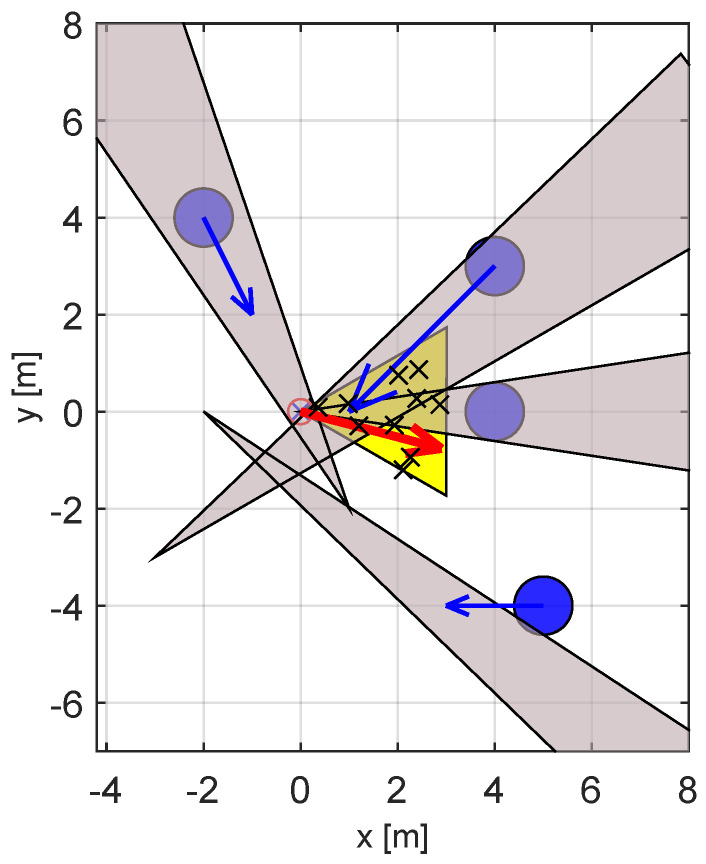
VO−type diagram of the crowded scenario. The robot is positioned in the origin and is denoted by a red circle, the goal position is at [8,0]. The yellow area represents the reachable available velocities, with the red arrow being the optimal velocity corresponding to the fastest solution. The blue disks represent the obstacles with their velocities, while the gray triangles are the corresponding VOs. The x-marks distributed within the area of reachable velocities are the initial population of the GAVO variants. The fact the majority of the yellow area is covered by the VOs shows that this is a “difficult” scenario.

The second series of our experiments considered a “crowded” environment, as shown in Figure 10. Note that this time there are four obstacles, with three dynamic obstacles that are heading roughly toward the current position of the robot. As the figure shows, the feasible set of velocities is almost completely covered by the VOs, making this a difficult scenario. The current location of the robot actually falls within one of the VOs, thus the robot needs to move in order to avoid colliding with one of the mobile obstacles. Finally, the goal position, at location [8,0], is obstructed by one of the obstacles, thus in the initial position the target is not visible to the robot.

We run a series of experiments for the four GAVO variants, with different values for the population size N (20, 50, and 100) and the GAP parameter (5 and 10). The results are listed in Table 1. In this table, *Max Gen.* is the maximum number of generations that can be executed during the maximum cycling time of 0.1 s. *Best Gen.* is the first generation from which the fitness value is not changing during the algorithm—a value marked as *best fitness*. The best time is the time needed to reach this value.

The four different recombination methods were compared in this situation as well. Table 1 represents the results using different parameters in the genetic algorithm. Strategy means the actual recombination strategy that was executed. ’Max Gen.’ means the number of the maximum generation that could be executed during the cycling time (0.1 s). ’Best Gen.’ is the first generation, from which the fitness value is not changing during the algorithm. Best time means the time interval needed to reach the best result and the best fitness means the best fitness value that could be reached in the algorithm using the different strategies.

Sometimes, the GAVO-1D recombination method cannot reach the best solution in real-time, e.g., N=20, GAP=10 (reaching the beast fitness value in 0.1485 s), or using the parameters N=50, GAP=5 (resulting the beast fitness value in 0.1526 s). The best solution can be reached considering the least number of generations using the GAVO-2D recombination method with parameters of N=100, GAP=5, resulting in the best fitness value in 5 generations. The fastest solution can also be reached considering the previous parameter set, resulting in a solution of 0.0133 s. Comparing all of the methods, the best fitness value is 0.67, which can be reached using the GAVO-1D method with parameters of N=100, GAP=10. To sum up, the result of the best fitness values comparing the different parameters in different recombination methods are quite close to each other and usually, they can be reached in real-time (except in some cases which were presented).

**Figure 11:**
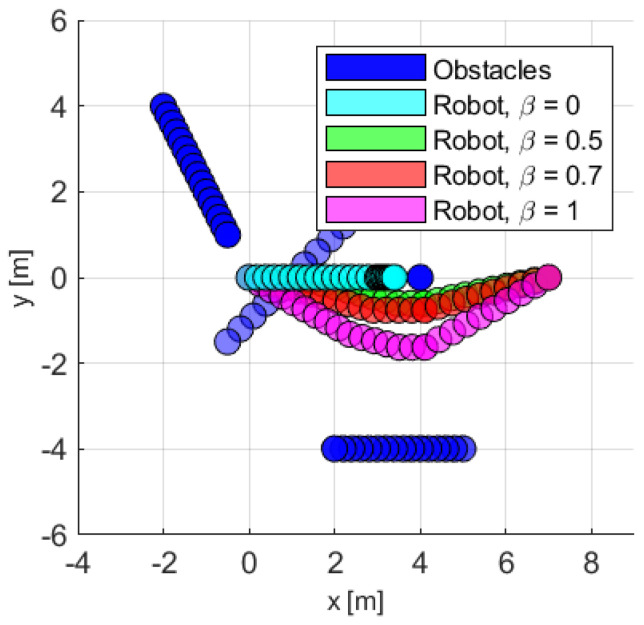
The successive locations of the robot (starting from $p_{A}={\left(0m,0m\right)}^{T}$) and obstacles outlining the paths taken by them during the scenario. Blue: obstacles ($B_{1}$ is a static obstacle at $p_{B1}={\left(4m,0m\right)}^{T}$. $B_{2}$ moves from ${\left(-2m,4m\right)}^{T}$ to ${\left(-0.5m,1m\right)}^{T}$. $B_{3}$ moves from ${\left(4m,3m\right)}^{T}$ to ${\left(-0.7m,-1.45m\right)}^{T}$. $B_{4}$ moves from ${\left(5m,-4m\right)}^{T}$ to ${\left(2m,-4m\right)}^{T}$.). Cyan: the robot using VO with the TG heuristic. Green: the robot using VO with the MV heuristic, identical with GAVO using β=1. Red: the robot using GAVO with β=0.7. Magenta: robot using GAVO with β=0. The target position is $p_{g}={\left(9m,0m\right)}^{T}$.

Finally, it is of interest to compare the trajectories generated by the different GAVO algorithm variants. Figure 11 shows the successive locations generated by two baseline heuristics and several GAVO variations. The paper introducing the VO concept also introduced two heuristic techniques TG and MV, which are widely used baselines in the literature. The TG (to goal) heuristic (in cyan in the figure) finds the safest path from the ones that directly head toward the goal. In this scenario, the goal is obstructed by a static obstacle, thus the robot will pick a trajectory that gradually comes to a stop in front of the static obstacle and never reaches the goal. The MV (maximum velocity) heuristic, in green in the figure, finds a strategy that allows the robot to move with the fastest velocity, possibly not directly in the direction of the goal. This trajectory reaches the goal; however, it takes a risky trajectory where the robot reaches a zero distance from the static obstacle. In this scenario, any sensor noise can lead to a collision. The same trajectory would be obtained by the GAVO method with parameter β = 1. By varying the β parameter, we can trade off the speed and safety of the trajectory found by GAVO, with β = 1 (magenta) corresponding to the safest trajectory, and β = 0.7 an intermediate solution balancing speed and safety.

### 5.3. Discussion

The variations of the GAVO technique achieve good performances (in many situations finding optimal solutions) and can also provide real-time algorithms, provided that the hyperparameters are carefully chosen. This led us to answer in the affirmative the question asked in the title of this paper, an answer that has changed since the original velocity obstacle paper when, realistically, only single-shot heuristics could be considered for real-time deployment. Our current ability to deploy techniques such as GAVO in real time is due to two separate developments: the increase in performance of computational devices that can be deployed in a robot (at least three to four orders of magnitude) and the better understanding of genetic algorithms and their dependence on representation and hyperparameters. For instance, Figure 7 shows the eight-fold computational cost difference between the best-performing vs. worst-performing GAVO variants. Undoubtedly, there are further possibilities for performance increase.

A possible weakness of the proposed approach is due to the fact that, as with any stochastic search technique, it offers only probabilistic guarantees about reaching a specific performance level in a given amount of time. Nevertheless, this weakness is partially compensated by the anytime nature of the algorithm and the elitism in the selection strategy that allows the algorithm to return at any given moment a good although possibly not optimal solution.

## 6. Conclusions

In this paper, we investigated whether genetic algorithms can serve as real-time path planners for obstacle avoidance for mobile robots. Historically, genetic algorithms were considered computation-heavy techniques that are primarily suitable for offline deployment. We proposed a technique called GAVO, which combines genetic algorithms with the velocity obstacle method, and investigated several variants of the chromosomal representation and parametrization. Through a series of carefully measured experiments, we investigated both the performance of the algorithms as well as their computational costs. We found that with a careful choice of parameters and representation, GAVO can run in real-time on a current generation of hardware. Some of these improvements are due to the higher computing capabilities of current hardware, which allows the genetic algorithms to perform a wider exploration of the solution space. At the same time, we found that the appropriate choice of representations and crossover techniques can make a very large (eight-fold) difference in the computation time needed to reach the optimal solution.

We also believe that the technique can benefit from further research in more optimal representations and efficient computation techniques. Our future work will include finding more efficient representations that accelerate the search for optimal solutions. We also plan to investigate techniques to guide the search toward more efficient solutions, by seeding the genetic algorithms pool with solutions obtained through one-shot heuristics and balancing the exploration/exploitation balance. Another technique we will explore is the possibility of a parallel implementation of the GAVO model on appropriate hardware. 

## Figures and Tables

**Table 1:** Results of experiments on the crowded scenario using different GAVO variants, population sizes N and GAP parameters.

*N*	GAP	Variant	Max Gen.	Best Gen.	Best Time [s]	Best Fitness
20	5	GAVO-MUT	45	13	0.0621	0.7000
20	5	GAVO-2D	97	7	0.0136	0.7000
20	5	GAVO-1D	103	38	0.0427	0.6800
20	5	GAVO-Polar	100	15	0.0209	0.7000
20	10	GAVO-MUT	51	17	0.0536	0.7000
20	10	GAVO-2D	69	10	0.0257	0.7000
20	10	GAVO-1D	80	124	0.1485	0.6800
20	10	GAVO-Polar	73	16	0.0228	0.7000
50	5	GAVO-MUT	42	12	0.0402	0.7000
50	5	GAVO-2D	42	8	0.0190	0.7000
50	5	GAVO-1D	55	76	0.1526	0.6900
50	5	GAVO-Polar	49	15	0.0340	0.7000
50	10	GAVO-MUT	36	14	0.0384	0.7000
50	10	GAVO-2D	32	9	0.0190	0.7000
50	10	GAVO-1D	38	28	0.0770	0.6900
50	10	GAVO-Polar	42	16	0.0374	0.7000
100	5	GAVO-MUT	31	12	0.0369	0.7000
100	5	GAVO-2D	32	5	0.0133	0.7000
100	5	GAVO-1D	31	14	0.0457	0.6800
100	5	GAVO-Polar	32	17	0.0517	0.7000
100	10	GAVO-MUT	27	11	0.0400	0.7000
100	10	GAVO-2D	28	15	0.0140	0.7000
100	10	GAVO-1D	23	7	0.0254	0.6700
100	10	GAVO-Polar	31	18	0.0577	0.7000

## Data Availability

Not applicable.

## Abbreviations

In this paper, we use the following notations:

VO	velocity obstacle shape
p	position vector
v	velocity vector
$v_{max}$	length of the maximum velocity vector
*r*	length of velocity vector
$T_{max}$	maximum time interval
$t_{min_{A,Bi}}$	minimum time at the filtering method when the agent and the obstacle are at the closest points to each other
$d_{max}$	maximum distance
$d_{min_{A,Bi}}$	minimum distance between the agent and the obstacle during their motion
GO	speed component of the fitness function
SA	safety component of the fitness function
θ	angle of a vector
Δθ	difference between the angle of the goal and the angle of the velocity vector
*f*	fitness function
β	parameter in the fitness function which shows the impact of the safety
κ	random parameter
